# Electrophysiological measures of resting state functional connectivity and their relationship with working memory capacity in childhood

**DOI:** 10.1111/desc.12297

**Published:** 2015-03-17

**Authors:** Jessica J. Barnes, Mark W. Woolrich, Kate Baker, Giles L. Colclough, Duncan E. Astle

**Affiliations:** ^1^ Medical Research CouncilCognition and Brain Sciences UnitCambridgeUK; ^2^ Oxford Centre for Human Brain ActivityUniversity of OxfordUK; ^3^ Department of Medical GeneticsUniversity of CambridgeUK

## Abstract

Functional connectivity is the statistical association of neuronal activity time courses across distinct brain regions, supporting specific cognitive processes. This coordination of activity is likely to be highly important for complex aspects of cognition, such as the communication of fluctuating task goals from higher‐order control regions to lower‐order, functionally specific regions. Some of these functional connections are identifiable even when relevant cognitive tasks are not being performed (i.e. at rest). We used magnetoencephalographic recordings projected into source space to demonstrate that resting state networks in childhood have electrophysiological underpinnings that are evident in the spontaneous fluctuations of oscillatory brain activity. Using the temporal structure of these oscillatory patterns we were able to identify a number of functional resting state networks analogous to those reported in the adult literature. In a second analysis we fused this dynamic temporal information with the spatial information from a functional magnetic resonance imaging analysis of functional connectivity, to demonstrate that inter‐subject variability in these electrophysiological measures of functional connectivity is correlated with individual differences in cognitive ability: the strength of connectivity between a fronto‐parietal network and lower‐level processing areas in inferior temporal cortex was associated with spatial working memory capacity, as measured outside the scanner with educationally relevant standardized assessments. This study represents the first exploration of the electrophysiological mechanisms underpinning resting state functional connectivity in source space in childhood, and the extent to which the strength of particular connections is associated with cognitive ability.

## Research highlights

- Magnetoencephalography (MEG) can be used to extract neurophysiological data from typically developing children, which can be used to explore functional connectivity at rest.
- At rest the temporal structure of neural oscillations can be used to decompose the developing brain into constituent networks.
- The temporally precise information in MEG can be fused with the spatially precise information in fMRI to explore electrophysiological connections with networks of interest.
- Across children, connections with a bilateral fronto‐parietal network at rest covary with the child's spatial working memory capacity as measured outside the scanner.

## Introduction

The development of methods capable of exploring neural activity at a systems level has opened a number of new research avenues, allowing researchers to go beyond defining the functions of single areas to investigate how and when activity is coordinated across brain areas. One such method is functional connectivity analysis, which includes investigating the temporal correlation of endogenously fluctuating activity between spatially discrete neuronal populations (Aertsen, Gerstein, Habib & Palm, 1989; Friston, Frith, Liddle & Frackowiak, 1993), with this coordination in activity potentially providing a basis for large‐scale communication between brain areas. In some cases these patterns of coordinated brain activity are apparent even at rest, in the form of resting state networks (RSNs). The fact that similar networks have been consistently identified across studies despite differing equipment and analysis protocols speaks to the robustness of these networks (van den Heuvel & Hulshoff Pol, 2010). Functional connections change extensively during childhood and one assumption is that these developing networks support the emergence of higher‐order cognitive functions (see Menon, 2013, for review). Exactly how aspects of the neurophysiological activity within emerging functional networks relate to cognitive mechanisms in childhood remains largely unexplored.

In childhood, RSNs have been studied using functional magnetic resonance imaging (fMRI), which measures down‐stream haemodynamic processes occurring in response to changes in neural metabolism. The RSNs observed in children using this technique (Thomason, Dennis, Joshi, Joshi, Dinov *et al*., 2011) broadly mirror those observed in adults (Greicius, Krasnow, Reiss & Menon, 2003; De Luca, Smith, De Stefano, Federico & Matthews, 2005; Damoiseaux, Rombouts, Barkhof, Scheltens, Stam *et al*., 2006). However, even by late childhood, networks associated with higher‐order cognitive functions, such as dorsal fronto‐parietal networks, can still be fragmented, composed of separate underlying components rather than behaving as a coordinated system as in adulthood (de Bie, Boersma, Adriaanse, Veltman, Wink *et al*., 2012).

Due to the dynamically fluctuating nature of the activity of these networks, it is necessary to consider the use of multiple methodologies to examine the rich neurophysiological basis of this inter‐regional coordination. One such methodology, which can be used to complement and expand upon the findings of fMRI, is magnetoencephalography (MEG). Like electroencephalography (EEG), which measures electrical currents conducted to the scalp and commonly used with developmental populations, MEG also noninvasively measures electrical activity. However, it does this by detecting magnetic fields produced by underlying electrical activity, which are not distorted to nearly the same degree by the scalp and skull. As a result, using source reconstruction techniques it is possible to transform the MEG sensor recordings into an estimate of the electrical activity of sources within the brain more reliably than with EEG recordings. Via source reconstruction, MEG therefore offers possibilities for exploring functional connectivity at a neurophysiological and anatomical level in source space. MEG offers comparable temporal resolution to EEG, imaging functional activity on millisecond timescales, and provides information about the frequency content of the neuronal oscillatory patterns. Therefore, unlike fMRI, MEG offers the ability to constrain connectivity analyses to particular frequencies of oscillation (the approach taken here), and to study rapidly changing or transient network structures (Baker, Brookes, Rezek, Smith, Behrens *et al*., 2014). An important difference, relative to using EEG, is that in this case we are not looking at event‐related changes in activity. Despite a number of researchers starting to use EEG to explore resting activity, the most common approach in using EEG to study developmental populations is to use its high temporal resolution to explore event‐related changes in oscillatory synchronization or derive event‐related potentials (ERPs, e.g. Astle, Harvey, Stokes, Mohseni, Nobre *et al*., 2014a). Instead, here we use spontaneous fluctuations in children's oscillatory brain activity, recorded while they are not performing any specific task (at rest) in the MEG scanner, to explore how dynamic patterns of activity across brain regions can become coordinated.

Because methods for non‐invasively measuring electrophysiological brain connectivity are in their infancy, studies relating functional connectivity to cognitive ability have hitherto largely relied upon functional connectivity fMRI (fc‐fMRI) methods. Evidence with this technique suggests that in adulthood particular functional connections at rest are related to cognitive ability (e.g. Stevens, Tappon, Garg & Fair 2012). The implication is that the variability across individuals in the strength of a functional connection measured at rest reflects intrinsic differences in the volume or efficiency of communication across that particular connection, thereby limiting the extent to which these regions can coordinate their action, and that this constrains ability during task performance. Even at rest, the organization and efficiency of a fronto‐parietal network is predictive of adults' working memory ability outside the scanner (Stevens *et al*., 2012). There have been very few studies exploring the relationship between RSNs and cognitive ability in childhood, although in one case results mirror the pattern of the adult literature: variability in connectivity between frontal and parietal regions is associated with cognitive ability, although not defined beyond IQ (e.g. Wu, Taki, Sato, Hashizume, Sassa *et al*., 2013). We can find only one study that has explored the relationship between functional connectivity and cognitive ability in childhood with MEG, and this was limited to analyses of the connectivity between MEG sensors, rather than between projected sources within the cortex (Ortiz, Stingl, Munssinger, Braun, Preissl *et al*., 2012). In the current study we related MEG measures of functional connectivity to short‐term and working memory performance (STM and WM, respectively) outside the scanner. There exists a large literature using well‐validated and educationally relevant assessments of individual differences in STM and WM in childhood (e.g. Alloway, Gathercole, Kirkwood & Elliott, 2009). These tasks typically recruit relatively long‐range and variable connections in adulthood, including fronto‐parietal control networks (e.g. Lepsien, Griffin, Devlin & Nobre, 2005), generating clear predictions for plausible associations between memory capacity and functional connectivity in childhood.

To summarize our approach: In this study we sought to take the first steps in using MEG to measure (a) the electrophysiological nature of functional brain connectivity in childhood, and (b) relate these new measures of functional connectivity to measures of STM and WM taken outside the scanner. To do this, first we tested whether it is possible to observe RSNs using the temporal structure of spontaneous patterns of oscillatory brain activity within the MEG signal, as measured in children at rest, as has recently been demonstrated in adults (Brookes, Woolrich, Luckhoo, Price, Hale *et al*., 2011). We aimed to benchmark these networks against similar networks produced using an fc‐fMRI analysis. Second, we combined the temporal precision of MEG with spatial information from fc‐fMRI; we extracted real‐time electrophysiological information within predefined canonical fMRI RSNs of interest, and identified connections between these networks and other neural systems. Third, we tested the hypothesis that individual differences in RSN activity assessed via MEG source‐based analysis can predict cognitive ability. In particular we applied a form of multiple regression (or a general linear model, GLM) to explore whether and how these electrophysiological connectivity measures would predict children's abilities in control demanding tasks, specifically those designed to tax STM/WM.

## Methods

### Participants

Thirty‐one children, aged between 8 and 11 (mean age = 119.2 months; standard deviation (*SD* = 11.3 months; 12 males) were recruited via local schools. We selected this age range as it is a phase during which complex span WM assessment scores are reliable, but cognitive abilities are still emerging and have yet to reach adult potential (Astle *et al*., 2014a). Moreover, we know that individual differences in children's WM abilities at this age are highly predictive of their educational attainment, meaning that we could explore the neurophysiological correlates of an ability that we can confidently describe as educationally relevant (Gathercole, Pickering, Knight & Stegmann, 2004). The only exclusion criterion was that children should not have a diagnosis of a developmental disorder or acquired neurological condition. All of the children had normal or corrected‐to‐normal vision. Parents provided written informed consent and the study was approved by the University of Cambridge Psychology Research Ethics Committee.

### Cognitive assessments

We conducted an assessment of each child's STM and WM using a number of subtests from the Automated Working Memory Assessment (AWMA; Alloway, Gathercole, Kirkwood & Elliott, 2008). We assessed each child's verbal STM using a forward digit span procedure, and their verbal WM using a backwards digit span. We assessed each child's spatial STM using a dot matrix task, in which they had to retain a number of spatial locations and then report them in sequence. Each child's spatial WM was assessed using a spatial span task, in which they had to retain the locations of a sequence of dots for subsequent recall, while performing a series of mental rotations. Performance for each child on each task was then compared to that of a normalization sample, such that the age‐independent standardized scores could be established (with a mean of 100 and *SD* of 15). These standard scores were used to characterize our sample (see Results section). However, in our MEG analysis, we used raw scores in the GLM. This is important because the standardized scores from the AWMA are age standardized in whole‐year sections, meaning that children with the same raw score, but who are only a few days apart in age, could have very different age‐standardized scores. The age standardization also alters the distribution of the scores. Instead we used the raw scores in our GLM, but included a separate regressor of age in months. We could then explore the effects of memory capacity while controlling for effects of age. In our analysis we used two composite memory scores, one verbal and one spatial (see Results section). We will subsequently refer to these two measures as verbal WM and spatial WM, respectively, although it is important to note that each contains both one STM and one WM span measure.

**Figure 1:**
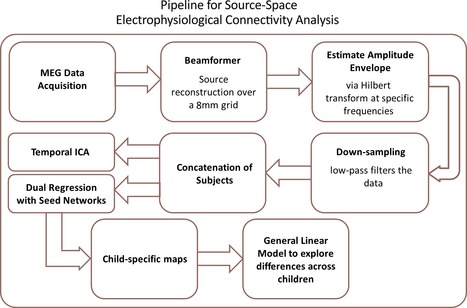
A flowchart of our analysis pipeline. The details of each step are described in the main text.

The subsequent MEG analysis is described in the flow chart shown in Figure 1. The corresponding steps are described in detail in the following sections.

#### MEG data acquisition and pre‐processing

MEG data were acquired with a high‐density whole‐head VectorView MEG system (Elekta‐Neuromag, Helsinki, Finland), containing a magnetometer and two orthogonal planar gradiometers at 102 positions (306 sensors in total), housed in a magnetically shielded room. Data were sampled at 1 kHz, and signals slower than 0.01 Hz were filtered out. A 3D digitizer (Fastrack Polhemus Inc., Colchester, VA, USA) was used to record the positions of five head position indicator (HPI) coils and 50–100 additional points evenly distributed over the scalp, all relative to the nasion and left and right preauricular points. We also attached an electrode to each wrist to measure the pulse, and we attached bipolar electrodes to obtain horizontal and vertical electrooculograms (HEOG and VEOG). Head position was monitored throughout the recoding using the HPI coils. Particularly small children were seated on a booster seat to ensure that their head was optimally positioned within the scanner helmet. For the resting state data acquisition, children were instructed to close their eyes, let their mind wander and not think of anything in particular for the duration of the scan. Data acquisition lasted 9 minutes. All children were monitored by video camera throughout the scan, and no child reported having fallen asleep during the scan.

External noise was removed from the MEG data using the signal space separation method, and adjustments in head position within the recording were compensated for using the MaxMove software, both implemented in MaxFilter version 2.1 (Elekta Neuromag). The MaxFilter software works by mathematically transforming the data to a set of virtual sensors. This is possible because the software has very accurate location measurements of both the MEG sensor array and the subject's head position. Because of our continuous recording of each child's head position, we could check at each sample of the MEG recording that the child's head was accurately transformed to the standard reference frame – thereby controlling for any within‐session head movements. Transforming the data to a set of virtual sensors also allows for the removal of noise emanating from outside the scanner. This signal space separation method acts to suppress any activity that does not stem from virtual channels within the helmet. The data were also down‐sampled to 250 Hz at this stage. The continuous data were visually inspected and any short sections with large signal jumps were removed. This is important, because if left in the data these large signal jumps could have a detrimental influence on the time‐frequency decomposition. A sensor‐space temporal independent component analysis (ICA) was then used to remove artefacts arising from blinks, saccades and pulse‐related cardiac artefacts using a combination of metrics and manual inspection. The temporal ICA was conducted separately for each subject using fastICA run on the sensor space data, and then the time course of each independent component (IC) was correlated with the time course of the VEOG, HEOG and cardiac channels, respectively. Components correlating more than Pearson *r *=* *0.1 with any of these were subsequently removed from the data. Components dominated by 50 Hz noise were removed to reduce the impact of interference from mains electricity.

#### MEG source reconstruction (beamformer)

Each subject's MEG data were co‐registered to a standard MNI template using the digitized scalp locations and fiducials via an iterative closest point algorithm using SPM8 (http://www.fil.ion.ucl.ac.uk/spm/). Prior to beamforming the data were bandpass‐filtered to focus only on the slower frequencies (theta: 4–7 Hz; alpha: 8–13 Hz; and beta: 14–30 Hz); previous work has shown that these slower frequencies are better for exploring functional connections with MEG, and increase discrimination between spurious and genuine connectivity (Luckhoo, Hale, Stokes, Nobre, Morris, Brookes *et al*., 2012). For each subject, source space activity was estimated at every vertex of an 8 mm grid covering the entire brain, using a linearly constrained minimum variance beamformer (Van Veen, van Drongelen, Yuchtman & Suzuki, 1997). The beamformer combined information from both the magnetometers and planar gradiometers while taking into account the reduced dimensionality of the data introduced by the signal space separation algorithm (Woolrich, Hunt, Groves & Barnes, 2011). Beamforming constructs a set of spatial filters which are applied to the sensor data to reconstruct the signal at each grid point throughout the brain, with the aim of achieving unit bandpass response at the grid point while minimizing the variance passed from all other locations. The process can be repeated across all grid locations to achieve a whole‐brain source reconstruction.

#### Amplitude envelope estimation, down‐sampling and concatenation

Once we had a dataset that contained source‐projected oscillatory data, the amplitudes of those oscillations were estimated via computation of the absolute value of the analytic signal, which was found using a Hilbert Transform. In essence, this yields an estimate of instantaneous signal amplitude at each voxel, at each of our frequencies of interest. The envelope time series for every voxel was then effectively low‐pass filtered by dividing each envelope time course into 1s windows and averaging within those windows (Brookes *et al*., 2011, who also used the same frequencies of interest as we used here). Both beamformer‐weights‐normalized and non‐beamformer‐weights‐normalized envelopes were estimated for use in the subsequent group‐level (general linear model) analysis (Luckhoo, Brookes & Woolrich, 2014). Spatial smoothing was also applied to the down‐sampled envelope estimates (FWHM 5 mm).

Once we had down‐sampled amplitude envelopes for all source space voxels and all subjects, we temporally concatenated the beamformer‐weights‐normalized envelopes across all children to produce one continuous data set that contained all children's data, adjoined end‐to‐end.

Following these steps, we analysed our MEG data in two ways. First, we used a temporal ICA to explore the presence of RSNs in our group of children, without providing any prior spatial information as to the likely constitution of the networks. Second, we identified three particular networks of interest, using spatial information from an independent fc‐fMRI dataset, and extracted electrophysiological information from those networks using a dual regression technique. Using this combined fc‐fMRI / MEG analysis we could then explore functional connections between other brain systems and these networks on the basis of this electrophysiological information. Finally, the whole‐brain maps produced for each child using this technique could be entered into a GLM in order to explore whether differences in connectivity at rest are associated with individual differences in WM capacity.

#### Temporal ICA

Our first type of analysis, the temporal ICA, was performed using fastICA, in which the data were reduced to 25 dimensions using a principal component analysis (PCA), and then divided into 25 temporally independent time series (Luckhoo *et al*., 2012). An ICASSO algorithm carried out 30 iterations of the fastICA (Hyvärinen, 1999) before clustering the results, in order to overcome the issues due to random initializations in the ICA. These 25 independent time series were each converted into covariance spatial maps by estimating the covariance between each independent time course and the down‐sampled amplitude envelope time course, concatenated across subjects, associated with each voxel. That is, the maps show the extent to which each voxel covaries with a particular component, and therefore each map provides the spatial distribution of each component. In essence, this ICA method identifies brain regions with a similar temporal structure to their oscillatory envelopes, implying that their activity is coordinated at rest.

#### Using fc‐fMRI seed networks and a general linear model (GLM)

**Figure 2:**
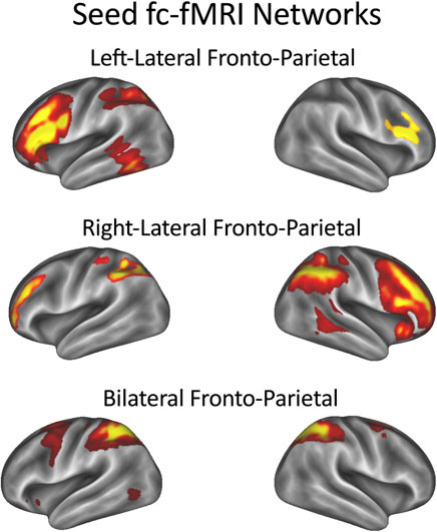
Three networks of interest selected a priori from the adult literature. These particular spatial maps were taken from a study using a spatial ICA with fc‐fMRI (Smith et al., 2012).

Here we tested whether variability in some particular seed RSNs would relate to individual differences in memory capacity using a multi‐subject group GLM. We used an independent set of 20 canonical RSNs as a basis set for this analysis from a recent fc‐fMRI study of RSNs in adulthood (Smith, Miller, Moeller, Xu, Auerbach *et al*., 2012; see Figure 2). The advantage of choosing networks a priori from this independent data set is that it utilizes the stronger spatial information in fc‐fMRI, providing a well‐identified standard set of RSNs. Within this set of canonical RSNs we then only looked for differences over subjects in the left and right lateral fronto‐parietal and bilateral fronto‐parietal networks. We chose these networks a priori because they represent our best approximation of those cortical networks particularly responsible for cognitive control in adulthood. It is possible that better expression of, and / or communication with, adult end‐state cognitive control networks is associated with enhanced STM/WM in childhood. We therefore tested whether differences in these networks, or in the areas that communicate with them, might explain individual differences in memory capacity across children.

To extract subject‐specific maps for each of the fc‐fMRI networks, we performed a MEG‐adapted dual regression (DR_MEG_) analysis (Luckhoo, 2014), analogous to the dual regression approach developed for use in fMRI data (Filippini, MacIntosh, Hough, Goodwin, Frisoni *et al*., 2009). In the first stage of DR_MEG_, we performed a spatial regression of the fc‐fMRI network maps on the concatenated beamformer‐weights‐normalized envelopes to yield concatenated network time courses. In the second stage, we broke these concatenated time courses into subject‐specific blocks. For each subject, we performed a temporal regression of the network time course segment from the non‐beamformer‐weights‐normalized downsampled envelopes. This gave a spatial map for each RSN that is specific to each subject, but critically has an unbiased estimate of the true variance of activity for that RSN, which is essential for all subsequent multi‐subject statistics (Luckhoo *et al*., 2014). The result of the dual regression was that for each subject and candidate network we obtained a whole‐brain map that corresponded to each voxel's strength within that network.

We used our two non‐standardized composite WM scores as two subject‐wise regressors. Each of these was de‐meaned and then used in a subject‐wise GLM, where they were regressed onto the subject‐wise data for each of our candidate networks. This regression was repeated separately for every voxel. For each measure we tested for significant effects in the raw scores over and above a control regressor of age in months – i.e. we measured the variance explained by each cognitive ability regressor over and above that explained by age. We also included gender as a regressor in the model, because previous research has shown that this too can account for some differences across children (Reiss, Abrams, Singer, Ross & Denckla, 1996; Speck, Ernst, Braun, Koch, Miller *et al*., 2000). In short, we used the following linear model: connectivity at each voxel equals beta1 * Verbal WM + beta2 * Spatial WM + beta3 * Age + beta4 * Gender + noise. The beta values from this model were then used to explore the neural impact of each of our cognitive measures, while controlling for the effect of age. The outcome was that for each of our networks we had a whole‐brain dataset in which we had estimated the linear contribution of both spatial and verbal WM scores (as indexed by the corresponding beta values from the model), while controlling for age. We adopted a standard procedure for testing the hypothesis that there are no significant clusters of voxels. We identified clusters of contiguous voxels where the output of the voxel‐wise GLM was greater than *t *=* *2.3. This value is essentially arbitrary, since it is the subsequent permutation process that actually tests for significance. We chose this value because it is that most routinely used in functional imaging experiments (e.g. http://fsl.fmrib.ox.ac.uk/fsl/fslwiki/Cluster). Once we had identified the size of these clusters we conducted a sign‐flipping permutation procedure to produce a null distribution, using 5000 permutations. We were then able compare the size of each result to this null distribution thereby identifying the relative alpha level and producing a *p*‐value. This non‐parametric permutation approach has a number of advantages relative to more traditional approaches to significance testing with electrophysiological data: first, it makes no a priori assumptions about when or where effects are likely to be apparent; second, this approach accounts for multiple comparisons over space and time, which can result in an uncontrolled false positive rate if uncorrected (Kilner, 2013). To summarize: the result of this GLM analysis is that it identifies areas within the candidate networks whose inclusion positively or negatively covaries with individual differences in WM, and that these results are fully corrected for multiple comparisons at a whole‐brain level.

## Results

### Cognitive assessments

Our sample of children had the following standardized mean scores: Verbal STM – 113.5 (*SD* = 18.2); Verbal WM – 104.9 (*SD* = 16.4); Spatial STM – 105.5 (*SD* = 17.3); Spatial WM – 111.6 (*SD* = 14.9). We subsequently averaged the two spatial and two verbal measures because they were highly correlated across the 31 children [Pearson correlations: Verbal – *r*(31) = 0.703, *p *<* *.001; Spatial – *r*(31) = 0.473, *p *=* *.007]. We used a dependent‐samples Fisher *r*‐to‐*z* transform to test whether these two correlations differed significantly, and they did not [*z*(31) = 1.344, *p *=* *.1789]. These composite verbal and spatial memory scores were later used to explore the relationship between functional connectivity and WM capacity.

### Temporal ICA result

The temporal ICA produced 75 temporally independent components (25 per time frequency band). These were then converted into spatial maps, as described in the Methods Section, and a number of these matched closely with typically reported RSNs identified in MEG and fMRI in adults (Brookes *et al*., 2011). We employed the following procedure for identifying these matches. First we calculated the spatial cross‐correlation, using the FSL tool fslcc, between our MEG maps within each frequency band and the set of candidate MRI maps previously derived using fc‐fMRI (Smith *et al*., 2012). These cross‐correlations provide information about the degree of spatial overlap between the maps derived using standard procedures with fc‐fMRI, and those derived using the dynamic temporal structure of oscillatory brain activity in MEG. The MEG map which best matched the fc‐fMRI counterpart was selected. The corresponding cross‐correlation value was then converted into a non‐standardized *z* statistic, such that we could calculate a *p*‐value for the cross‐correlation.

We also tested the significance of our correlations with a second non‐parametric permutation approach, which controlled for the effect of choosing the best match over 75 maps. In each case we selected the best matching MEG map, and randomly shuffled the allocation of voxel‐wise values across the brain. The process was repeated 75 times, in each case spatial smoothing was applied (5 mm FWHM), to produce a set of 75 random MEG maps. The spatial cross‐correlation between each of these and the candidate fc‐fMRI map was then calculated and the map with the strongest match chosen – just as we had with our real MEG maps. This process was repeated 5000 times in order to produce a null distribution, which characterized the strength of spatial cross‐correlation that would be expected when having 75 random maps and selecting the best match. We could then compare the spatial cross‐correlation from our real map to this distribution to test whether the strength of association is greater than we would have expected by chance (*p*_perm_ < .05).

**Figure 3:**
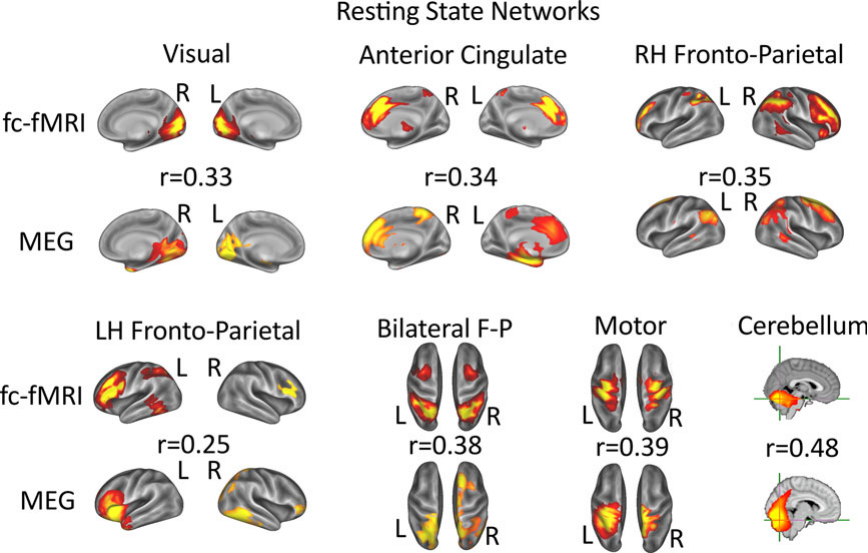
Spatial maps of RSNs derived from our source space‐projected MEG data, alongside the associated spatial map from fc‐fMRI (Smith et al., 2012). The r‐values correspond to cross‐correlations between the two maps for each modality. The MEG maps show covariance between the temporally down‐sampled Hilbert envelope at each voxel and the time course of that particular temporally independent component. These maps include an early visual component (theta band), the Anterior Cingulate Cortex (alpha band), a right‐hemisphere fronto‐parietal network (beta band), a left‐hemisphere fronto‐parietal component (beta band), a bilateral fronto‐parietal network (theta band), a sensory motor component (beta band) and the Cerebellum (beta band). For visualization purposes, each MEG spatial map is thresholded at 2.3. Each fc‐fMRI map is thresholded at 5. Where the component maps are of cortical areas they are depicted on cortical renderings, whereas where they include non‐cortical areas (i.e. the Cerebellum) they are shown on whole‐brain images.

Following this approach, in the beta band we were able to identify a component with separate nodes in the right hemisphere frontal and parietal cortex [*r *=* *0.35, *z *=* *0.3654, *p *<* *.001, *p*_perm_ < .001], and another component with a distinct node in the left hemisphere frontal cortex [*r *=* *0.25, *z *=* *0.2554, *p *<* *.001, *p*_perm_ < .001]. In the alpha band we observed a component with a node in anterior cingulate cortex [*r *=* *0.34, *z *=* *0.3541, *p *<* *.001, *p*_perm_ < .001]. We also observed a component in the theta band which included a more superior portion of the parietal and frontal lobes [*r *=* *0.38, *z *=* *0.4001, *p *<* *.001, *P*_perm_ < .001]. In addition to these different components incorporating different parts of the frontal lobe, we also identified a cerebellar component (beta band) [*r *=* *0.48, *z *=* *0.5230, *p *<* *.001, *p*_perm_ < .001], an early visual component (theta band) [*r *=* *0.33, *z *=* *0.3428, *p *<* *.001, *P*_perm_ < .001] and a sensorimotor component (beta band) [*r *=* *0.39, *z *=* *0.4118, *p *<* *.001, *p*_perm_ < .001]. Figure 3 shows the MEG maps and their fc‐fMRI counterparts. From this we can see that some of the networks, while present, are poorly defined in children relative to adults (Brookes *et al*., 2011). This is especially the case for the left hemisphere fronto‐parietal network. There are also notable absences – the default mode network and the posterior parietal cortex component. In the former case this may not be surprising given that prior fMRI work has shown that this can be fragmented at this age (de Bie *et al*., 2012). In the latter case there were significant matches, but these were also matches with other networks that incorporated portions of posterior parietal cortex, implying that we did not have the spatial precision with this data set to distinguish this component from others. As such the matches that we report above represent the strongest unique matches. It is important to note that any apparent differences between children and adults (Brookes *et al*., 2011) would need to be tested directly with a single dataset that contains both age groups.

### Combined fc‐fMRI /MEG and GLM result

**Figure 4:**
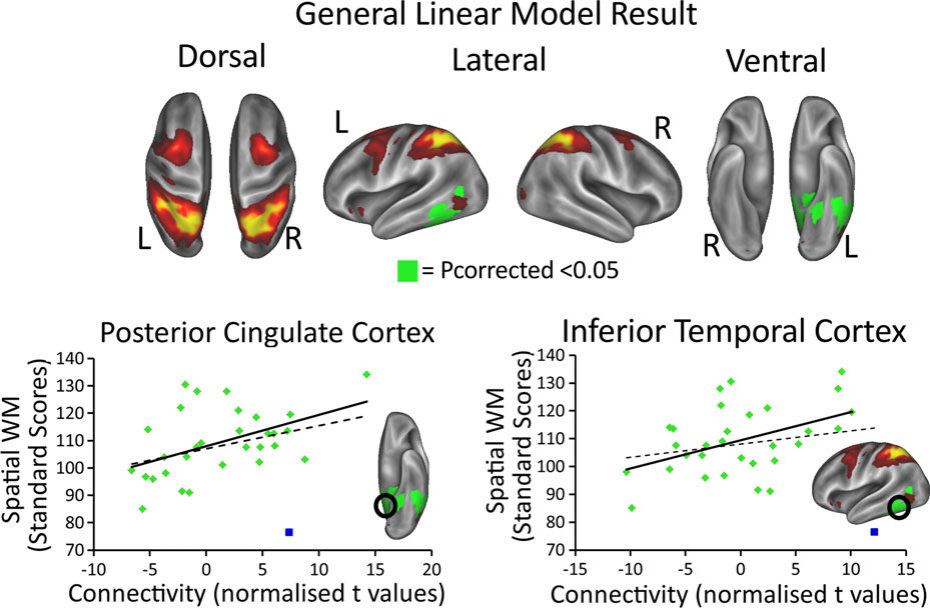
The results of the voxel‐wise GLM procedure. The green area shows a region of the bilateral fronto‐parietal network that covaried significantly with a child's spatial WM; the greater the connectivity between this region and the rest of the network, the greater the child's composite spatial WM score (p_corrected_ < .05). The green region spans a section of visual cortex, stretching from the posterior cingulate cortex to area IT. To illustrate the relationship between connectivity in these areas this figure also shows the correlation between connectivity (indexed by normalized T scores) in posterior cingulate cortex or area IT and age standardized spatial WM scores (Pearson r = 0.48, and r = 0.45, respectively). In both cases one child was left out of the correlation because their composite spatial WM score was an outlier, being more than two standard deviations below the mean. This child is the blue data point in each scatter plot. In each scatter plot the regression line was calculated with (solid line) and without (dashed line) this child. Note that the child was only designated as an outlier for the purposes of plotting this correlation – for the actual temporal ICA and GLM analysis the child was included.

In addition to exploring the presence of RSNs using the temporal ICA, without any spatial constraint, we also defined three a priori seed networks of interest for a comparison across individuals. These were three fronto‐parietal networks derived using a spatial ICA in fMRI data (Smith *et al*., 2012). We used the dual regression approach described in the Methods section to fuse this spatial information with the electrophysiological activity from the MEG. We could then calculate whole‐brain maps for each child that expressed the degree of connectivity with these networks. These maps were then submitted to a GLM to test for any connectivity differences that mirrored individual differences in WM capacity. The connectivity pattern between a lower‐level processing area and one of these networks in the alpha band significantly mirrored individual differences in spatial WM. The strength of the correlation between the nodes of the bilateral fronto‐parietal network (shown in Figure 2) and a cluster of voxels that included a section of inferior left temporal cortex (close to area IT), stretching to the tip of the posterior cingulate cortex, significantly predicted a child's spatial WM capacity, as measured outside the scanner. This effect survived our whole‐brain correction for multiple comparisons (*p*_corrected_ = .0280). This can be seen in Figure 4. To illustrate the reason for this significant effect we have plotted the correlation between connectivity and age‐standardized spatial WM capacity at two voxels, one in posterior cingulate cortex (MNI = −2, −60, 24) and the other in area IT (MNI = −50, −48, −14), which can be seen in Figure 4. Another reason for plotting these correlations is to confirm our GLM analysis, which does not use age‐standardized scores per se, but uses raw scores and controls for age using a separate regressor of age in months. When plotting these correlations, one particular child was marked as an outlier because their spatial WM score of 76.5 was more than two standard deviations (13.44) below the mean (108.21) of the rest of the sample. In Figure 4 the regression line is plotted both with (solid line) and without (dashed line) this child included. Note that the plotting of this correlation does not affect the child's inclusion in the actual analysis – the temporal ICA and GLM both include this child. No other effects survived the whole‐brain correction for multiple comparisons.

An interesting issue that we return to in the Discussion section is the lack of effect of verbal WM. This was included in the model in just the same way as spatial WM, but the parameter corresponding to this did not significantly relate to individual differences in connectivity in our seed networks. To check whether this resulted from being included in a model alongside spatial WM we repeated our analysis pipeline but including verbal WM in a model without spatial WM. However, this produced the same result – there was no significant relationship between individual differences in connectivity with our three seed networks and verbal WM.

## Discussion

The current study aimed to take the first steps in using source space‐projected MEG data to explore the electrophysiological underpinnings of resting state functional connectivity in childhood. We used the temporal structure of the neural oscillations to parcellate the developing brain into temporally distinct networks, which were identifiable as a number of RSNs that are widely reported in adults. We then combined the spatial information from an fc‐fMRI analysis with the dynamic electrophysiological information from MEG to examine how this connectivity was related to WM capacity in childhood. We defined fronto‐parietal networks in fc‐fMRI and used the temporal structure of the neuronal oscillations in these networks to explore their variable coordination with other areas in the developing brain. Electrophysiological coordination between one particular bilateral fronto‐parietal network and a set of lower‐level processing areas covaried with a child's spatial WM capacity, as measured outside the scanner. These findings confirm the usefulness of MEG in extracting richly informative neurophysiological data for functional connectivity analysis, and that this information can be used to explore the intrinsic processing limits that may be associated with differences in WM capacity.

In this study we demonstrated the feasibility and utility of using MEG to investigate resting state functional connectivity in childhood. Methods for studying invasive electrophysiological recording in humans and non‐human primates have demonstrated the informative data that can be obtained by directly measuring dynamic patterns of neural activity across brain regions (see Engel, Fries & Singer, 2001, for a review). However this direct recording approach does not currently allow the researcher to explore networks across multiple broad systems, and is not suitable for use with most human volunteer populations. Source‐projected MEG data obtained in this study allowed us to investigate this spatially and temporally rich electrophysiological information using a measure of neural activity at a systems level. We used temporal ICA as a blind source‐separation technique, decomposing the mixture of signals into distinct components. Importantly, we did not specify any spatial information for this part of our analysis, with the spatial distribution of the networks being an emergent property of the underlying temporal pattern of the electrophysiological activity. In addition to assaying the electrophysiological nature of the underlying networks, the method is relatively assumption‐free and not at all dependent upon a choice of regions of interest. We demonstrated the validity of this approach in our first analysis, using it to successfully derive RSNs similar to those reported in the fc‐fMRI literature.

The use of a beamformer in the current study, as a means of projecting sensor‐level recordings into source space, marks another substantial difference relative to previous developmental studies of resting state activity. Projecting sensor data into source space allowed us to explore RSNs at the level of cortical neuroanatomy and investigate networks in the spatial dimension. The beamformer acts to suppress spatially separate but temporally correlated sources – that is, the beamformer is biased against identifying highly temporally correlated sources. This latter characteristic helps this analysis pipeline mitigate a common problem in performing connectivity analyses on electrophysiological data – signal leakage, the mischaracterization of one source as two connected sources (Schoffelen & Gross, 2009). This might intuitively make beamforming seem like an extremely conservative tool for exploring functional connectivity. However, our analysis depended upon the temporally down‐sampled Hilbert *envelope* of the raw source‐projected signal, rather than the raw oscillatory signal itself. This means that connected sources can be out of phase with one another, ruling out signal leakage as a cause, but their envelopes could still be highly correlated, meaning that these connections can still be detected using this analysis pipeline.

### The electrophysiological underpinnings of RSNs in childhood

Our MEG‐based investigation identified left and right hemisphere fronto‐parietal, anterior cingulate, sensorimotor, visual, frontal and cerebellar components in resting state. These networks show a significant similarity to RSNs previously observed in both developmental and adult fc‐fMRI studies. The purpose of this exploratory analysis of the oscillatory data was to demonstrate, for the first time, the feasibility of using MEG to measure the electrophysiological basis of resting state connectivity in childhood. A limitation of the current study is that we only explore these networks within a relatively narrow age range. While this enables us to explore individual differences without gross structural differences in neuroanatomy, this does limit the conclusions that we can draw about the development of these electrophysiological mechanisms. While we observed apparent differences between the RSNs of children and adults, future studies are now needed to build upon this proof‐of‐concept and test directly for these developmental differences (e.g. Cheour, Imada, Taulu, Ahonen, Salonen *et al*., 2004). While there will be further technical challenges to overcome in comparing across children, adolescents and adults of large age differences, an added benefit of this approach is that it will enable us to tease apart developmental and individual differences in connectivity, which is theoretically very important. A second limitation is that this is not a fully multi‐modal dataset. There may be sensitivity differences between the technique that we apply and others. To address this fully we need to acquire a full multi‐modal dataset, with both BOLD‐based and MEG measures, in order to provide a full comparison of methods across the age span.

There are many interesting questions pertaining to the electrophysiological basis of functional connectivity in childhood which could now be explored using the method described; these include the impact of development, differences across populations of interest, and the impact of interventions. The purpose of this part of our analysis was to provide the first demonstration that MEG data can be usefully applied in future to address these questions. We then apply this approach to addressing one particularly relevant question – how do neurophysiological mechanisms of connectivity mirror differences across children in well‐validated, educationally relevant, measures of cognition?

### Inter‐subject variability in functional connectivity and cognitive ability in childhood

As networks supporting advanced cognitive processing are still undergoing maturation during childhood and are highly variable between individuals, it is of interest to investigate how this network variability might relate to cognitive ability in childhood. Cognitive or executive control is responsible for the optimization and regulation of cognitive processes, and is a pertinent example of why functional connectivity is so important for typical brain functioning. Individuals frequently encounter tasks in daily life that demand a considerable degree of cognitive control, such as when employing WM, and this requires them to organize and maintain multiple pieces of information, often while ignoring distraction or when at the limits of their maintenance capacity (Baddeley & Hitch, 1974; Astle, Nobre & Scerif, 2012b). The deployment of control during these tasks depends upon functional connections that can communicate evolving task goals to those areas of the brain that are processing the relevant sensory input. However, there have been almost no studies of resting state connectivity differences and cognition in childhood and none using MEG in source space, a situation we sought to redress. Our combined fc‐fMRI and MEG analysis revealed that variability in functional coordination between a bilateral fronto‐parietal network and lower‐level processing areas, including inferior temporal cortex, covaries with spatial WM capacity. This relationship cannot be explained by differences in strategy or motivation, since the children were at rest, nor can it be explained by basic differences in vasculature across the children, since we used measures directly related to neural activity. In short, we identified a core physiological characteristic of this network that mirrors a child's spatial WM ability.

The specific network implicated here includes bilateral superior parietal cortex and middle frontal gyri (the frontal eye fields (FEF)). These areas are typically associated with both covert and overt spatial attention processes (e.g. Schwartz, Vuilleumier, Hutton, Maravita, Dolan *et al*., 2005; Taylor, Nobre & Rushworth, 2007), but have also been implicated in a number of other cognitive control mechanisms such as task‐switching (Astle, Nixon, Jackson & Jackson 2012a). Recent research with non‐human primates has shown that electrophysiological signals in FEF can be reflected in visual processing areas, including V4 and area IT, when attentional control mechanisms are recruited (Gregoriou, Gotts, Zhou & Desimone, 2009). The current study demonstrated the utility of MEG recordings for measuring these electrophysiological mechanisms in childhood, and moreover, that the degree of intrinsic connectivity between this control network and these sensory processing areas is predictive of spatial WM capacity across children. Our findings might therefore reflect the network dynamics that play a key role in successful cognitive performance during a WM task; that is, the intrinsic functional connection between frontal and parietal regions and regions within inferior temporal cortex at rest forms a critical pathway for communication, and stronger connectivity at rest may subsequently result in improved communication during WM performance. However, this final causal mechanism remains to be tested, and a necessary complementary step would be to combine these functional network analyses with a task‐positive dataset (e.g. Astle, Luckhoo, Woolrich, Kuo, Nobre *et al*., 2014b).

It is noteworthy that we could not identify a similar intrinsic connection predictive of children's verbal WM. There are several possible reasons for this. First, there could be less consistency in the mechanisms children use to solve verbal WM tasks. For example, some children may rely simply upon their basic capacity for retaining verbal material, whereas others may rely upon chunking strategies that lend themselves well to retaining verbal material in serial order (Burgess & Hitch 1999). This increased variability in mechanism may make it more difficult to identify significant relationships with intrinsic connectivity, especially when applying a relatively conservative correction for multiple comparisons. Second, it could be that we simply did not include the correct seed networks in our analysis. We chose seed networks based upon their consistent role in control‐demanding tasks (e.g. Lepsien *et al*., 2005), but it is possible that connections with other more language‐specialized areas (Smith, Jonides & Koeppe, 1996) would be more closely linked to verbal WM. A final related possibility is that the diverse areas responsible for language processing, which might be recruited for verbal WM, are less detectible using MEG.

In summary, the current study used the richly informative electrophysiological information obtained through MEG to characterize network connectivity at rest in children, and demonstrated that physiological differences in communication between discrete cortical areas and a fronto‐parietal network at rest are significantly related to individual differences in spatial WM.
